# The additive effects of the *TM6SF2 E167K* and *PNPLA3 I148M* polymorphisms on lipid metabolism

**DOI:** 10.18632/oncotarget.18474

**Published:** 2017-06-14

**Authors:** Lizhen Chen, Shuixian Du, Linlin Lu, Zhonghua Lin, Wenwen Jin, Doudou Hu, Xiangjun Jiang, Yongning Xin, Shiying Xuan

**Affiliations:** ^1^ College of Medicine and Pharmaceutics, Ocean University of China, Qingdao, Shandong, China; ^2^ Department of Gastroenterology, Qingdao Municipal Hospital, Qingdao, Shandong, China; ^3^ Medical College, Qingdao University, Qingdao, Shandong, China; ^4^ Central Laboratories, Qingdao Municipal Hospital, Qingdao, Shandong, China

**Keywords:** nonalcoholic fatty liver disease, additive effect, TM6SF2, PNPLA3, bayesian network

## Abstract

There is a genetic susceptibility for nonalcoholic fatty liver disease (NAFLD). To examine the role of genetic factors in the disease, a Bayesian analysis was performed to model gene relationships in NAFLD pathogenesis. The Bayesian analysis indicated a potential gene interaction between the *TM6SF2* and *PNPLA3* genes. Next, to explore the underlying mechanism at the cellular level, we evaluated the additive effects between the *TM6SF2 E167K* and *PNPLA3 I148M* polymorphisms on lipid metabolism. Hepa 1-6 cells were transfected with a control vector or with overexpression vectors for TM6SF2/PNPLA3-wild type, TM6SF2-mutant type, PNPLA3-mutant type, or TM6SF2/PNPLA3-mutant type. Commercial kits were used to measure triglyceride and total cholesterol levels in each of the five groups. The mRNA and protein expression levels of sterol regulatory element-binding transcription factor 1c and fatty acid synthase were analyzed using real-time PCR and western blotting. The triglyceride and total cholesterol contents were significantly different among the groups. The triglyceride and total cholesterol contents and the sterol regulatory element-binding transcription factor 1c and fatty acid synthase mRNA and protein expression levels were significantly higher in the TM6SF2/PNPLA3-mutant type group than in the TM6SF2-mutant type group or the PNPLA3-mutant type group. The *TM6SF2 E167K* and *PNPLA3 I148M* polymorphisms may have additive effects on lipid metabolism by increasing the expression of sterol regulatory element-binding transcription factor 1c and fatty acid synthase.

## INTRODUCTION

Nonalcoholic fatty liver disease (NAFLD) is the most common chronic liver disease in the world, affecting 13.48–31.79% of the general population [1]. In recent years, the incidence of NAFLD has been higher than that of viral hepatitis and alcoholic liver disease. NAFLD can be histologically categorized as simple fatty liver, which is usually considered a benign lesion, or as nonalcoholic steatohepatitis [2]. The latter pathological process can dramatically confer increased risks of liver cirrhosis and hepatocellular carcinoma [3] and is characterized by liver inflammation and hepatocyte injury in addition to hepatic steatosis with or without fibrosis [2, 4].

There is a genetic susceptibility for NAFLD [5]. Accordingly, the role of genetic factors in NAFLD pathogenesis has been gaining greater attention. Recent studies, including a pilot study by our group, have shown that the *TM6SF2 E167K* [6–8] and *PNPLA3 I148M* [9–11] polymorphisms are both associated with the development of NAFLD. Interestingly, Kozlitina and colleagues observed that inhibition of TM6SF2 significantly reduced the expression of PNPLA3, a protein that plays an important role in triglyceride synthesis [6]. Furthermore, Goffredo *et al.* determined that the *TM6SF2 E167K*, *PNPLA3 I148M*, and *GCKR rs1260326* single nucleotide polymorphisms had a joint effect in determining intrahepatic fat accumulation in a cohort of obese children, including Caucasians, African Americans, and Hispanics [12]. These results indicated that the *TM6SF2* and *PNPLA3* polymorphisms might have additive effects in regulating lipid metabolism. However, the underlying molecular mechanism remains unclear.

The present study evaluated the additive effects of the *TM6SF2 E167K* and *PNPLA3 I148M* polymorphisms on lipid metabolism using bioinformatics approaches and then further explored the underlying mechanism at the cellular level.

## RESULTS

### The bayesian network and potential interaction between the *TM6SF2* and *PNPLA3* genes

**Figure 1:**
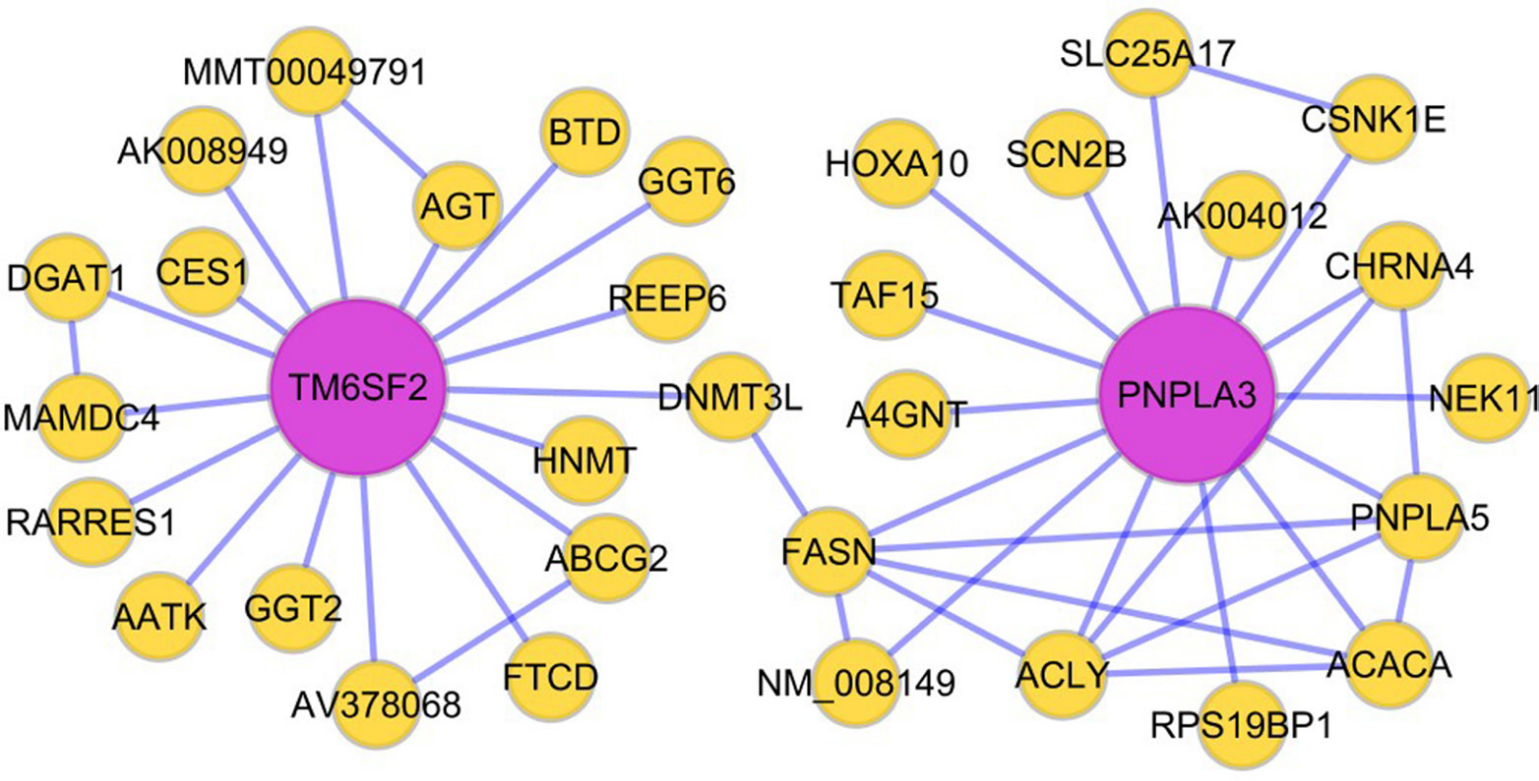
Hypothesized Bayesian network indicating linkage between the *TM6SF2* and *PNPLA3* genes The lines in the network represent transcriptional regulation between genes. The *TM6SF2* and *PNPLA3* genes may interact with each other through *DNMT3L* and *FASN*. Abbreviations: DNMT3L: DNA (cytosine-5)- methyltransferase 3-like; FASN: fatty acid synthase.

We performed bioinformatics analysis and data mining, and ultimately constructed a Bayesian network to model NAFLD pathogenesis. Table 1 describes the relevant source papers used to construct the Bayesian network, including research articles with liver expression data from both mice and humans. Figure 1 shows a visualization of a potential gene interaction in the Bayesian network focusing on the *TM6SF2* and *PNPLA3* genes, which are two important regulators of liver fat metabolism. Our data demonstrate that multiple genes related to lipid metabolism are connected with *TM6SF2* and *PNPLA3*. Based on the network shown in Figure 1, we hypothesized that the *TM6SF2* and *PNPLA3* genes may interact with each other through DNA (cytosine-5)-methyltransferase 3-like (*DNMT3L*) and *FASN*.

**Table 1:** The liver expression data of mouse and human and construction of the bayesian network

Tissue	Species	eSNP data	Co-expr. modules	Bayesian networks	Dataset
Liver	Human	yes	yes	yes	427 individuals [13]
	Human	yes	yes	no	1,008 obese patients [14]
	Mouse	-	yes	yes	C57BL/6J × A/J mouse cross [15]
	Mouse	-	yes	yes	C57BL/6J × C3H ApoE –/– mouse cross [16, 17]
	Mouse	-	yes	yes	C57BL/6J × C3H wild-type mouse cross [13]
	Mouse	-	yes	yes	C57BL/6J × BTBR Lepob mouse cross [18]

### Effects of the *TM6SF2 E167K* and *PNPLA3 I148M* variants on triglyceride and total cholesterol contents in hepa 1-6 cells

**Figure 2:**
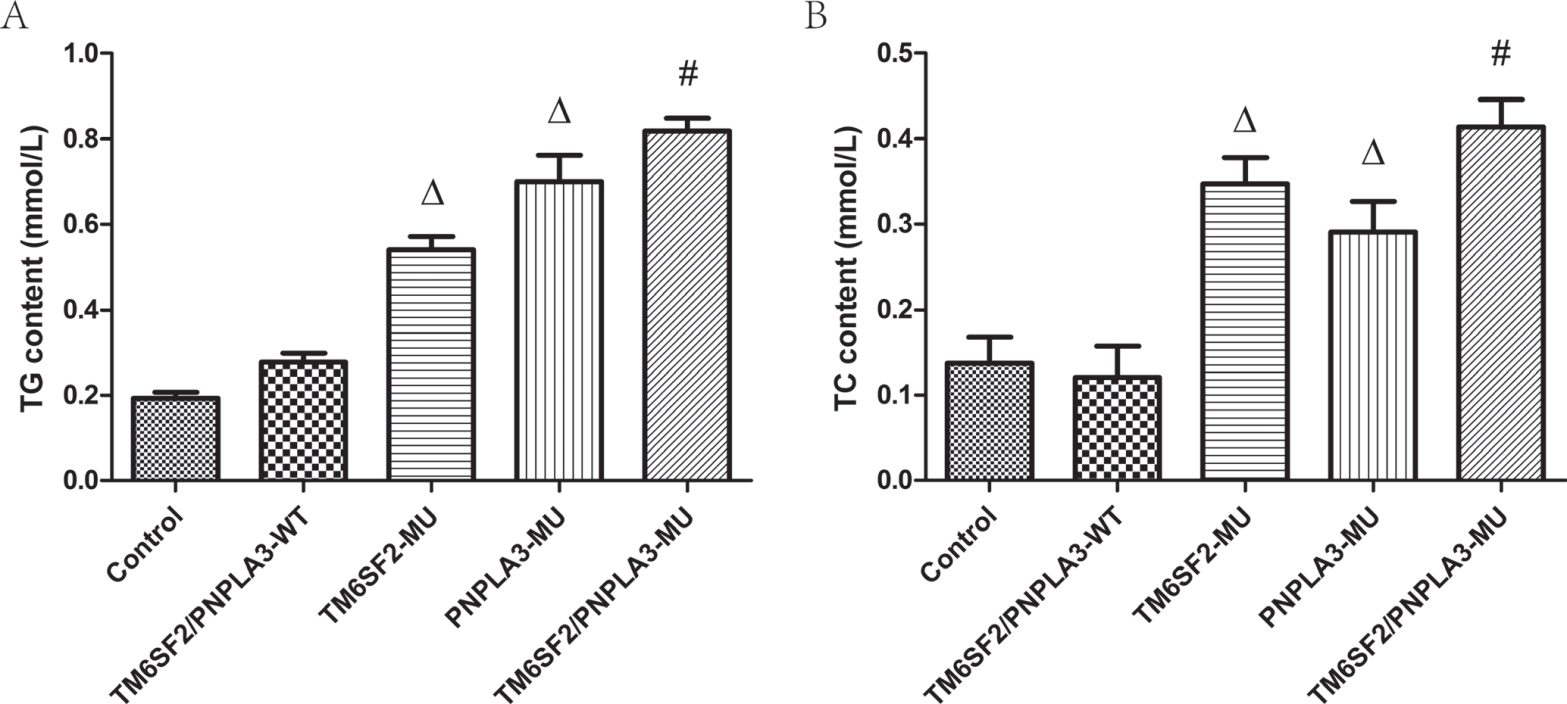
Effects of the *TM6SF2 E167K* and *PNPLA3 I148M* variants on triglyceride and total cholesterol contents The *TM6SF2 E167K* and *PNPLA3 I148M* variants increased triglyceride (TG) and total cholesterol (TC) contents. ^Δ^*P* < 0.01 *vs.* TM6SF2/PNPLA3-WT group, ^#^*P <* 0.01 *vs.* either the TM6SF2-MU group or the PNPLA3-MU group.

Figure 2 shows the triglyceride and total cholesterol contents for the five groups. According to the one-way ANOVA, there were differences in both the triglyceride (F = 169.48, *P* < 0.01) and total cholesterol (F = 44.79, *P* < 0.01) contents among the groups. Further LSD *t*-tests revealed that the triglyceride and total cholesterol contents of both the TM6SF2-MU group and the PNPLA3-MU group were higher than both the control group and the TM6SF2/PNPLA3-WT group (all *P* < 0.01). Moreover, significant differences in the triglyceride (*P* < 0.01) and total cholesterol (*P* = 0.035) contents were observed between the TM6SF2/PNPLA3-MU group and the TM6SF2-MU group. Similarly, there were differences in both the triglyceride (*P* = 0.002) and total cholesterol (*P* = 0.001) contents between the TM6SF2/PNPLA3-MU group and PNPLA3-MU group.

### Effects of the *TM6SF2 E167K* and *PNPLA3 I148M* variants on the expression of genes involved in triglyceride metabolism

**Figure 3:**
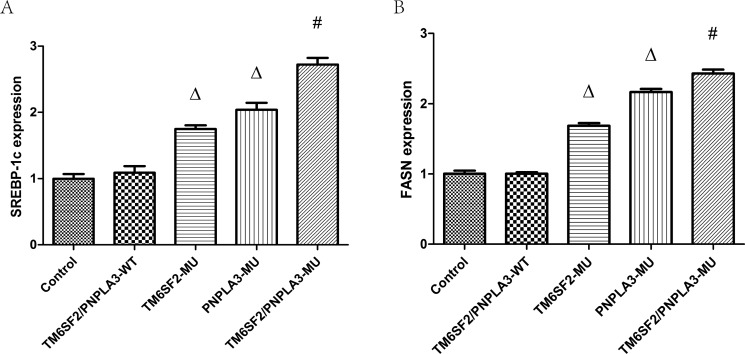
Effects of the *TM6SF2 E167K* and *PNPLA3 I148M* variants on *SREBP-1c* and *FASN* mRNA expression levels The *TM6SF2 E167K* and *PNPLA3 I148M* variants significantly upregulated the *SREBP-1c* and *FASN* mRNA expression levels. ^Δ^*P* < 0.01 *vs.* TM6SF2/PNPLA3-WT group, ^#^*P <* 0.01 *vs.* either the TM6SF2-MU group or the PNPLA3-MU group. Abbreviations: SREBP-1c: sterol regulatory element-binding transcription factor 1c; FASN: fatty acid synthase.

To investigate how the *TM6SF2 E167K* and *PNPLA3 I148M* variants mechanistically affect lipid content, we determined and analyzed levels of SREBP-1c and FASN mRNAs and proteins, which are involved in hepatic lipid synthesis and metabolism. Our data revealed that the expression of SREBP-1c and FASN mRNAs were significantly different among the five groups (F_SREBP-1c_ = 194.64, *P* < 0.01; F_FASN_ = 696.21, *P* < 0.01). As shown in Figure 3, the expression levels of both SREBP-1c and FASN mRNA were higher in the TM6SF2-MU group and the PNPLA3-MU group than in the TM6SF2/PNPLA3-WT group (all *P* < 0.01). Furthermore, the SREBP-1c and FASN mRNA expression levels in the TM6SF2/PNPLA3-MU group were significantly higher than both the TM6SF2-MU group and the PNPLA3-MU group (all *P* < 0.01).

**Figure 4:**
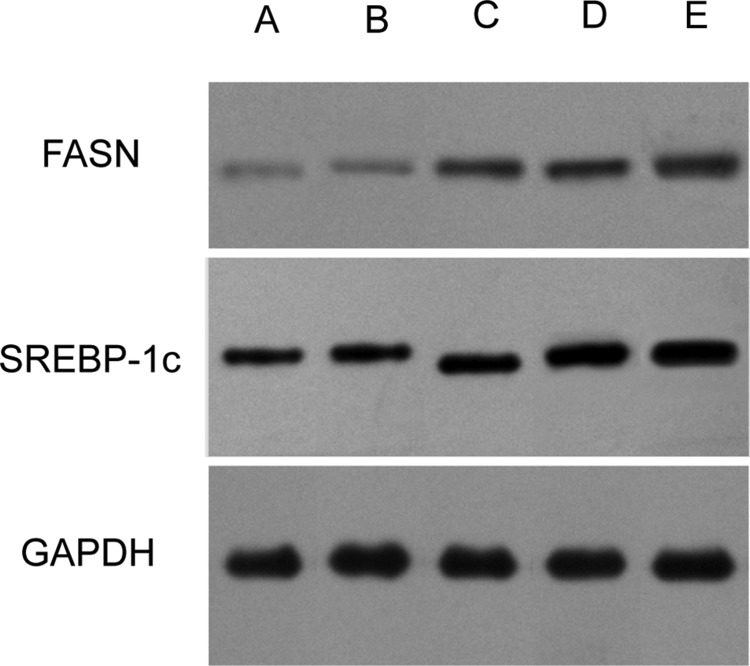
Effects of the *TM6SF2 E167K* and *PNPLA3 I148M* variants on SREBP-1c and FASN protein expression levels (**A**) the TM6SF2/PNPLA3-control group; (**B**) the TM6SF2/PNPLA3-wild type group; (**C**) the TM6SF2-mutant type group; (**D**) the PNPLA3-mutant type group; (**F**) the TM6SF2/PNPLA3-mutant type group. Abbreviations: SREBP-1c: sterol regulatory element-binding transcription factor 1c; FASN: fatty acid synthase; GAPDH: glyceraldehyde 3-phosphate dehydrogenase.

Consistent with the RT-PCR results, the western blotting analysis (Figure 4) revealed that the SREBP-1c and FASN protein levels were also significantly different among the five groups (F_SREBP-1c_ = 474.64, *P* < 0.01; F_FASN_ = 509.54, *P* < 0.01). As expected, the mutant forms of *TM6SF2 E167K* and *PNPLA3 I148M* caused a substantial increase of the levels of SREBP-1c and FASN proteins compared to the wild type (all *P* < 0.01). In addition, our data demonstrated that the SREBP-1c and FASN protein levels in the TM6SF2/PNPLA3-MU group were significantly higher than in both the TM6SF2-MU group (*P* SREBP-1c < 0.01, *P* FASN < 0.01) and the PNPLA3-MU group (*P* SREBP-1c = 0.005, *P* FASN < 0.01). Together, the results suggested that the *TM6SF2 E167K* and *PNPLA3 I148M* variants might increase hepatic lipid content by increasing SREBP-1c and FASN levels.

## DISCUSSION

The aim of this study was to investigate whether there are additive effects of the *TM6SF2 E167K* and *PNPLA3 I148M* polymorphisms on lipid metabolism. The major finding of our research is that the *TM6SF2 E167K* and *PNPLA3 I148M* variants may increase hepatic lipid content by increasing the expression of SREBP-1c and FASN.

*TM6SF2* and *PNPLA3* are two important genes that were identified as conferring susceptibility to NAFLD in a genome-wide association study and an exome-wide association study of multiethnic, population-based cohorts [6, 9]. Both are involved in very low-density lipoprotein (VLDL) secretion and lipid droplet remodeling. The human *TM6SF2* gene is located on chromosome 19 and encodes a protein of 351 amino acids [19]. The TM6SF2 protein is an EXPERA domain P-containing enzyme homolog, which was predicted to possess similar catalytic activity as sterol isomerases according to a computational protein sequence analysis [20]. Mahdessian *et al.* demonstrated that the TM6SF2 protein is predominantly located in both the endoplasmic reticulum (ER) and the ER-Golgi intermediate compartment of hepatocytes in a protein subcellular localization study [19]. Several studies provided evidence that TM6SF2, which is highly expressed in the liver and small intestine [6, 21], can be regarded as a “switch” mediating VLDL secretion [6, 19, 22]. The *PNPLA3* gene encodes a polypeptide chain of 481 amino acids and is located on the long arm of chromosome 22 [23]. PNPLA3 is also highly expressed on the ER and in lipid membranes of metabolically relevant tissues (i.e., liver and adipose tissue) [24]. Pingitore and colleagues reported that the PNPLA3 protein, which has a conserved patatin domain, displays predominant triglyceride hydrolase activity and mild lysophosphatidic acid acyltransferase activity [23].

Various studies have established strong links between the *TM6SF2 E167K* polymorphism and the developmental and progressive stages of NAFLD [6–8]. Similarly, studies have demonstrated an association between the *PNPLA3 I148M* polymorphism and the development of NAFLD [9–11]. However, NAFLD is a complex disease influenced by many factors, including genetics, diet, and gut microbiota [5, 25]. Neither the *TM6SF2 E167K* polymorphism nor the *PNPLA3 I148M* polymorphism alone can fully account for the molecular genetic mechanisms of NAFLD or disease risk. Analysis of gene-gene interactions is essential to understand the etiology of complex diseases and to identify genes responsible for disease susceptibility. In the present study, we provided evidence that the *TM6SF2* and *PNPLA3* genes may interact with each other through *DNMT3L* and *FASN* in a Bayesian analysis. Moreover, the results of our cellular experiments showed that the TM6SF2/PNPLA3-MU group had significantly higher triglyceride and total cholesterol contents than both the TM6SF2-MU group and the PNPLA3-MU group. Our finding is consistent with those of recent studies [12, 26]. Wang *et al.* found that the *TM6SF2 E167K* and *PNPLA3 I148M* variants were the most important risk alleles for NAFLD and indicated that they had an additive effect on NAFLD in a Chinese cohort [26]. Together, these findings indicate that the *TM6SF2 E167K* and *PNPLA3 I148M* polymorphisms have a potential additive effect in NAFLD.

**Figure 5:**
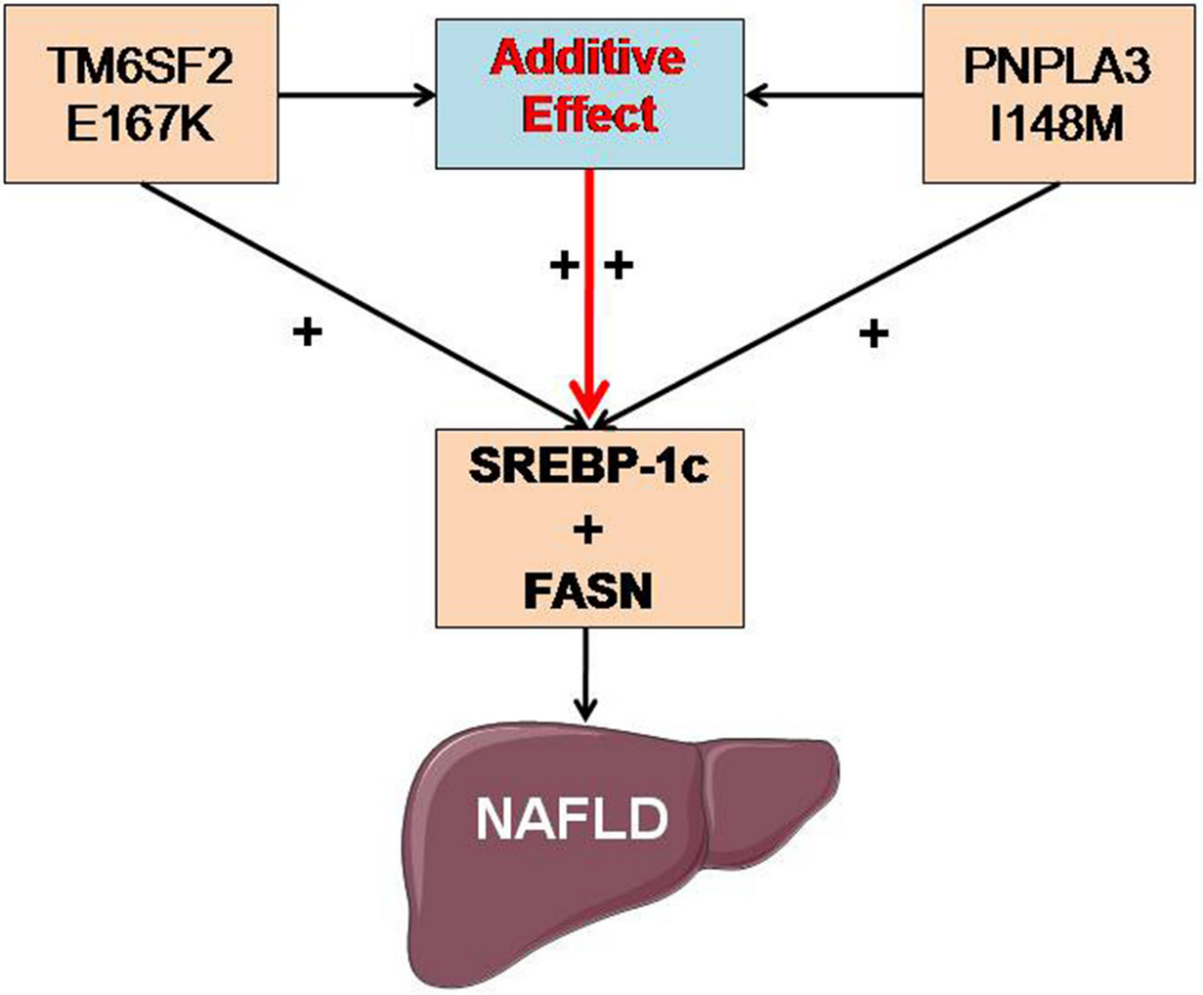
Potential molecular mechanism of the additive effects of the *TM6SF2 E167K* and *PNPLA3 I148M* polymorphisms on NAFLD The additive effects of the *TM6SF2 E167K* and *PNPLA3 I148M* polymorphisms on NAFLD may be associated with upregulating the expression of SREBP-1c and FASN. Abbreviations: SREBP-1c: sterol regulatory element-binding transcription factor 1c; FASN: fatty acid synthase; NAFLD: nonalcoholic fatty liver disease.

However, the specific underlying mechanism of the above additive effect has not yet been reported. To the best of our knowledge, this study is the first to provide data to understand the proposed additive effect mechanistically. SREBP-1c is a master modifier of fatty acid and triglyceride metabolism, and it is synthesized on the ER membrane in its precursor form [27–28]. As a metabolic regulator, SREBP-1c activates the transcription of target genes, including FASN, acetyl-CoA carboxylase, and stearoyl-CoA desaturase, and consequently, it enhances the synthesis of fatty acids and the accumulation of triglycerides [29]. Our data showed that SREBP-1c and FASN mRNA and protein expression levels were significantly increased in the TM6SF2/PNPLA3-MU group compared to both the TM6SF2-MU group and the PNPLA3-MU group. Considering these findings, we hypothesize that the additive effects of the *TM6SF2 E167K* and *PNPLA3 I148M* polymorphisms on NAFLD may be associated with upregulation of SREBP-1c and FASN expression (Figure 5). In the future, the role of *DNMT3L* in the additive effects of the *TM6SF2 E167K* and *PNPLA3 I148M* polymorphisms on lipid metabolism should be further elucidated. In addition, studies at the cellular and individual levels are required to understand the underlying mechanisms involved in this process further.

## MATERIALS AND METHODS

### Establishment of the bayesian network

The Bayesian algorithm is an attractive method that can provide formalism for statistical reasoning about partial beliefs under circumstances of uncertainty [29]. Here, a Bayesian network was constructed to model gene relationships in NAFLD pathogenesis according to previously established methods [30–31]. The network incorporated liver expression data from mice and humans. A Bayesian analysis was performed to explore potential interactions between the *TM6SF2* gene and the *PNPLA3* gene. These genes were reported to be important modifiers involved in the regulation of triglyceride metabolism.

### Hepa 1-6 cell culture

Hepa 1–6 cells were cultured in Dulbecco’s Modified Eagle’s Medium (DMEM) containing 10% fetal bovine serum (FBS; Hyclone, USA), 100 U/mL penicillin, and 100 μg/mL streptomycin (Gibco^®^, USA). The cells were incubated at a temperature of 37°C in a humidified atmosphere with 5% CO_2_. The cells were treated when they reached approximately 80% confluence.

### Construction of lentiviral vectors and transfection studies

The present study examined the following five groups: (1) the control group, (2) the TM6SF2/PNPLA3-wild type (WT) group (TM6SF2 EE + PNPLA3 II/IM), (3) the TM6SF2-mutant type (MU) group (TM6SF2 EK/KK + PNPLA3 II/IM), (4) the PNPLA3-mutant type (MU) group (TM6SF2 EE + PNPLA3 MM), and (5) the TM6SF2/PNPLA3-mutant type (MU) group (TM6SF2 EK/KK + PNPLA3 MM). The lentiviral plasmids for the experimental groups and control group were constructed by Shanghai Genechem Co., Ltd. For each group, the plasmids were transfected into 293T cells after the cells were cultured for 24 h in DMEM with 10% FBS. The 293T cells were incubated with the transfection complexes (expression plasmid, packaging plasmid, and transfection reagent) for 48–72 h. The lentivirus was then concentrated and transfected into Hepa 1–6 cells. The success of the transfection process in each group was validated by real-time polymerase chain reaction (RT-PCR) and western blotting, as well as by measuring the percentage of Hepa 1–6 cells with green fluorescent protein.

### Biochemical indicator assay

After transfection, the Hepa 1–6 cells were centrifuged, and the supernatants were collected. The concentrations of biochemical indicators (triglyceride and total cholesterol) were measured in each group using commercial kits following the manufacturer’s recommended instructions (Sigma-Aldrich, St. Louis, USA).

### Extraction of total RNA and quantitative RT-PCR

The expression levels of sterol regulatory element-binding transcription factor 1c (*SREBP-1c*) and fatty acid synthase (*FASN*), two factors associated with lipid metabolism, were analyzed using RT-PCR. Total RNA was isolated from the Hepa 1–6 cells using Trizol reagent (Invitrogen, USA) following the manufacturer’s protocol. Complementary DNA (cDNA) synthesis was performed using the RevertAid First Strand cDNA Synthesis Kit (Thermo Fisher: K1622, USA).

SYBR^®^ Green Realtime PCR Master Mix (Toyobo: QPK-201, Japan) was used for RT-PCR following the manufacturer’s instructions. For standardization, the glyceraldehyde 3-phosphate dehydrogenase (*GAPDH*) gene was used as an internal control. The specific primer sequences were designed and synthesized by the Springen Biotechnology and are shown in Table 2. The RT-PCR parameters consisted of denaturation at 95°C for 5 minutes, followed by 40 cycles of denaturation at 95°C for 15 s, annealing at 60°C for 20 s, and extension at 72°C for 40 s. The relative amounts of the above genes were obtained using the comparative threshold cycle (CT) method [32].

**Table 2:** Primer sequences for RT-PCR

Gene	Primer	**Sequences (5**′**–3**′**)**
SREBP-1c (163bp)	Forward Reverse	GCTCCCTAGGAAGGGCCGTA ACTTCACCTTCGATGTCGGTC
FASN (183bp)	Forward Reverse	CAGAGCAGCCATGGAGGAG GACAGGTCCTTCAGCTTGCC
GAPDH (202bp)	Forward Reverse	CATCTTCTTTTGCGTCGCCA TTAAAAGCAGCCCTGGTGACC

Abbreviations: RT-PCR: Real Time PCR; SREBP-1c: sterol regulatory element-binding transcription factor 1c; FASN: fatty acid synthase; GAPDH: glyceraldehyde 3-phosphate dehydrogenase.

### Western blotting

Total protein was extracted from the Hepa 1–6 cells using radioimmunoprecipitation assay buffer (Sigma-Aldrich, USA). The Bradford method was used to determine protein concentration following the manufacturer’s protocol, and the proteins were then frozen at –70°C until analysis. Antibodies against SREBP-1c (Santa Cruz Biotechnology, Inc., SC-13551, USA), FASN (Cell Signaling Technology, Inc., 3180S, USA), and GAPDH (Keygen Biotech, Co. Ltd., KGAA002, China) were used. The expression levels of the SREBP-1c and FASN proteins were normalized relative to the corresponding GAPDH (endogenous reference) level in each lane. The western blots were analyzed using Gel-Pro Analyzer Version 4.5 Software (Media Cybernetics, USA).

### Statistical analysis

Statistical significance was determined using one-way analysis of variance (ANOVA) followed by the least significant difference (LSD) *t*-test. The statistical comparisons among the groups were made using the Statistical Package for the Social Sciences (SPSS version 17.0, SPSS Inc., Chicago, USA) software. All continuous variables were expressed as the mean ± standard deviation (SD) of at least three independent experiments. *P* < 0.05 was considered statistically significant.

## CONCLUSIONS

The *TM6SF2 E167K* and *PNPLA3 I148M* polymorphisms may have additive effects on lipid metabolism and the development of NAFLD by upregulating the expression of SREBP-1c and FASN. Understanding how these additive effects contribute to lipid metabolism and NAFLD may help provide a new scientific basis for NAFLD pathogenesis and shed light on genetic predictors for early diagnosis and primary prevention of the disease. Further investigation is needed to elucidate these underlying mechanisms.
